# Inappropriate sinus tachycardia or…something else?

**DOI:** 10.1002/joa3.12250

**Published:** 2019-10-17

**Authors:** Lucian Muresan, Ronan Le Bouar, Stephane Greciano, Crina Muresan, Serban Schiau, Gabriel Cismaru, Jacques Levy

**Affiliations:** ^1^ Cardiology Department Emile Muller” Hospital Mulhouse France; ^2^ Cardiology Department Rehabilitation Hospital Cluj‐Napoca Romania; ^3^ Cardiology Department Louis Pasteur Hospital Colmar France

**Keywords:** focal atrial tachycardia, right atrial appendage

## Abstract

We report the case of a 45‐year old female patient with a past medical history of rheumatoid arthritis who presented to our cardiology department with a suspicion of inappropriate sinus tachycardia. Echocardiography showed a nondilated left ventricle with a preserved ejection fraction. A careful reinterpretation of her 12‐lead ECG reoriented the diagnosis toward an incessant atrial tachycardia. The diagnosis was confirmed by an electrophysiologic study performed with an electro‐anatomic mapping system, which identified the origin of the tachycardia at the level of the right atrial appendage. Radiofrequency ablation of the ectopic focus eliminated the tachycardia and improved the patient's symptoms.

## Graphical abstract

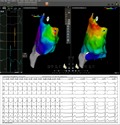

## 1 INTRODUCTION

Inappropriate sinus tachycardia is a condition characterized by an accelerated heart rate, usually above 100 bpm, without an identifiable cause. Patients suffering from inappropriate sinus tachycardia have a disproportionate acceleration of the heart rate in response to low‐intensity physical exercise. Establishing the correct diagnosis requires a careful history, physical examination, and laboratory tests. It is confirmed by a standard 12‐lead ECG, which excludes other causes of narrow QRS complex tachycardias, such as atrio‐ventricular nodal reentry tachycardia, atrio‐ventricular re‐entry tachycardia and atrial tachycardia. The differential diagnosis between inappropriate sinus tachycardia and an atrial tachycardia is often challenging, especially when the atrial tachycardia heart rate is slightly above 100 bpm, its origin is situated in the high right atrium conferring the P wave morphology similar to the sinus P wave morphology and when its character is incessant. An accurate diagnosis is important, since treatment options for atrial tachycardia include catheter ablation, which can eliminate the need for chronic antiarrhythmic administration.

## 2 CASE REPORT

**Figure 1:**
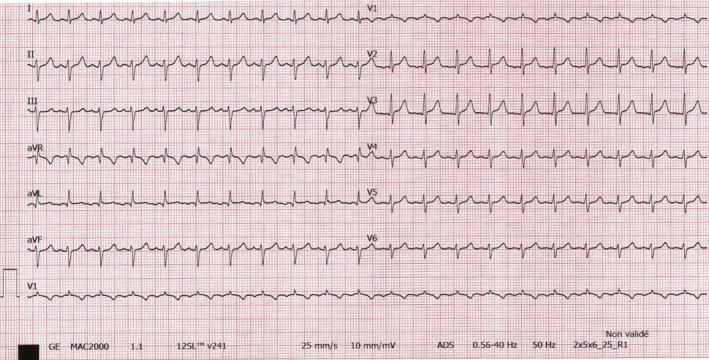
Twelve‐lead ECG showing a narrow QRS complex tachycardia with a heart rate of 126 bpm, left axis deviation, positive P waves in lead II, III, aVF, and DI (axis of + 77°), suggesting an origin in the superior part of the right atrium, compatible with sinus tachycardia

A 45‐year old female patient with a history of rheumatoid arthritis was addressed by her family physician for asthenia. Her cardiovascular history was relevant for inappropriate sinus tachycardia, a diagnosis established several months before. She was currently treated with Bisoprolol 2.5 mg, Metothrexate 7.5 mg/wk, folic acid supplements, Prednisone 6 mg/d, Risedonate 35 mg/wk, and Calcium + Vitamine D3 supplements. Her clinical examination was normal. The 12‐lead ECG showed a narrow QRS complex tachycardia with a heart rate of 126 bpm, the morphology of the P wave being positive in lead II, III, and aVF, positive in DI, suggesting an origin in the superior right atrium, compatible with sinus tachycardia (Figure 1).

Echocardiography revealed a nondilated left ventricle, with a LVEF of 62%, normal diastolic function, absence of significant valve disease, nondilated left atrium, absence of pulmonary hypertension, no pericardial effusion.

However, a closer look at the 12‐lead ECG allowed the identification of a negative P wave morphology in leads V1‐V2, with a predominantly positive P waves in the rest of the precordial leads, suggesting an origin at the level of the right atrial appendage,^1^ raising suspicion of an incessant ectopic atrial tachycardia with an origin at this level. An electrophysiologic study was programmed to confirm the diagnosis.

**Figure 2:**
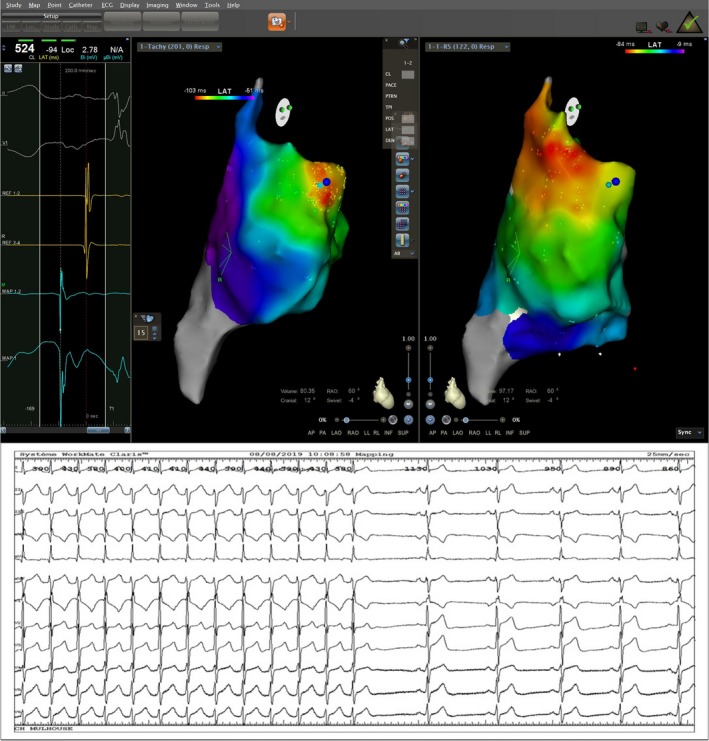
Left upper panel: Signal characteristics from the local successful ablation site: DII, V1, Coronary sinus catheter and Mapping catheter. Middle panel: CARTO 3 activation map of the right atrium during tachycardia in RAO 60° projection demonstrating a region of early endocardial activation (red zone) situated at the level of the right atrial appendage, with radial activation of the right atrium (color progression red → yellow → green → blue; see color legend in the upper part of the screen), suggesting a focal mechanism. Right upper panel: CARTO 3 activation map of the right atrium during sinus rhythm in RAO 60° projection showing the distance between the ectopic atrial tachycardia focus and the sinus node region, of 16 mm, explaining the subtle differences in the P wave morphology between the sinus rhythm and the atrial tachycardia. Lower panel: Twelve lead ECG at 25 mm/s showing tachycardia termination during radiofrequency ablation and restoration of the sinus rhythm

The electrophysiologic (EP) study was performed using the CARTO 3 electro‐anatomic mapping system. A first diagnostic ten‐polar catheter (Inquiry, Abbot®) was inserted via the femoral vein intro the coronary sinus and a second mapping/ablation roving catheter (Navistar ThermoCool Biosense Webster with a 3.5 mm irrigated tip) was placed in the right atrium and used to create an activation map during tachycardia. The EP study confirmed the presence of a focal atrial tachycardia originating from inside the right atrial appendage (Figure 2 left upper panel), with a likely increased automaticity mechanism (Video S1). Radiofrequency ablation at this level eliminated the tachycardia (Figure 2 lower panel).

A careful mapping of the right atrium during sinus rhythm demonstrated the origin of the arrhythmic focus at a distance of 15 mm from the sinus node region, explaining the subtle differences between the sinus P wave and the P wave during tachycardia (Figure 2 right upper panel).

The post ablation 24‐hour telemetry showed no atrial tachycardia recurrence. The patient was discharged from the hospital the next day with no antiarrhythmic drugs.

## 3 DISCUSSION

We present a rare case of ectopic atrial tachycardia originating from the right atrial appendage masquerading as inappropriate sinus tachycardia in a young female patient with rheumatoid arthritis.

The prevalence of atrial tachycardia originating from the right atrial appendage is reported to be between 0.6% and 8%.^1^, ^2^, ^3^ AT from RAA are commonly underdiagnosed and may sometimes lead to tachycardiomyopathy. Freixa et al^1^ tried to establish clinical, ECG, end electrophysiologic characteristics of atrial tachycardia arising from the right atrial appendage. They found that it is more common in males than in females (66% vs 38%; *P* = .013) and in younger patients (32 + 12.6 vs 55 + 13.2 years; *P* < .001). It is more frequently incessant (53% vs 16%; *P* < .001) and more likely to provoke left ventricular systolic dysfunction (27% vs 5%; *P* = .018). The authors identified a specific ECG pattern consisting of a negative P wave in leads V1‐V2 with a predominantly positive P waves in the rest of the precordial leads. This ECG pattern has a sensitivity of 100%, a specificity of 98%, a positive predictive value of 88%, and a negative predictive value of 100%. This pattern could also be identified in the present case.

The treatment of this type of tachycardia consists of antiarrythmic drugs^4^ or catheter ablation. The latter can be achieved with either radiofrequency^2^, ^3^ or cryotherapy.^5^ Radiofrequency ablation is very effective for this localization (100% vs 75%; *P* = .022) and usually there are no recurrences after a successful procedure (0% vs 8%; *P* = .31).^1^

This case report underlies the fact that an accurate interpretation of the 12‐lead ECG remains a cornerstone of both clinical and interventional cardiology, since subtle differences in its interpretation can orient the diagnosis toward a condition for which a radical treatment with a high success and low complication rate presently exists, in this case radiofrequency ablation.

## CONFLICT OF INTEREST

Authors declare no conflict of interests for this article.

## References

[1] Freixa X , Berruezo A , Mont L , Magnani S , Benito B , Tolosana JM , et al. Characterization of focal right atrial appendage tachycardia. Europace. 2008;10(1):105–9. 18077484 10.1093/europace/eum264

[2] Guo X‐G , Zhang J‐L , Ma J , Jia Y‐H , Zheng Z , Wang H‐Y , et al. Management of focal atrial tachycardias originating from the atrial appendage with the combination of radiofrequency catheter ablation and minimally invasive atrial appendectomy. Heart Rhythm. 2014;11(1):17–25. 24103224 10.1016/j.hrthm.2013.10.017

[3] Walsh EP , Saul JP , Hulse JE , Rhodes LA , Hordof AJ , Mayer JE , et al. Transcatheter ablation of ectopic atrial tachycardia in young patients using radiofrequency current. Circulation. 1992;86(4):1138–46. 1394921 10.1161/01.cir.86.4.1138

[4] Page RL , Joglar JA , Caldwell MA , Calkins H , Conti JB , Deal BJ , et al. 2015 ACC/AHA/HRS guideline for the management of adult patients with supraventricular tachycardia: a report of the American College of Cardiology/American Heart Association Task Force on Clinical Practice Guidelines and the Heart Rhythm Society. J Am Coll Cardiol. 2016;67(13):e27–e115. 26409259 10.1016/j.jacc.2015.08.856

[5] Amasyali B , Kilic A . Possible role for cryoballoon ablation of right atrial appendage tachycardia when conventional ablation fails. Tex Heart Inst J. 2015;42(3):289–92. 26175651 10.14503/THIJ-14-4238 PMC4473632

